# Incidence of Deep Venous Thrombosis and Sickle Cell Disease in Patients Undergoing Spinal Surgery in South Gujarat, India: A Prospective Observational Study

**DOI:** 10.5704/MOJ.2207.008

**Published:** 2022-07

**Authors:** HJ Menon, AP Khanna, YB Patel

**Affiliations:** Department of Orthopaedics, New Civil Hospital, Surat, India

**Keywords:** spinal surgery, sickle cell disease, deep vein thrombosis, prophylaxis

## Abstract

**Introduction:**

Our objective of this study was to assess the incidence of Deep Venous Thrombosis in patients including those with sickle cell disease who underwent spine surgery, and also to determine the association of Sickle Cell Disease as a clinical predictor for Deep Venous Thrombosis in spinal surgery patients.

**Materials and methods:**

All patients who underwent spinal surgery from January 2016 to October 2016 were included in this study. Detailed history, demographic data, physical findings, pre-operative haematological and radiological investigations were documented. All the patients underwent daily clinical evaluation for clinical signs of Deep Venous Thrombosis and also underwent a post-operative venous Doppler and D-dimer test.

**Results:**

Seventy-nine consecutive patients were included in the study with the mean age of 41 years. All patients had normal venous Doppler pre-operatively. A total of 2.5% patients had deep vein thrombosis in bilateral lower limbs while 2 patients (2.5%) had evidence of venous stasis but no thrombosis on Doppler ultrasound done post-operatively. Nine patients (11.4%) were sickle cell positive from which 4 patients showed evidence of Deep Venous Thrombosis or Venous Stasis. D-dimer was positive in 5 (8.3%) patients which included 4 patients with Sickle Cell Disease.

**Conclusion:**

This study concludes that Sickle Cell Disease is a risk factor for developing Deep Venous Thrombosis in patients undergoing spinal surgery. The study also concludes the effectiveness of mechanical prophylaxis in preventing Deep Venous Thrombosis and recommends pharmacological prophylaxis after assessing the risk profile or positive D-dimer test.

## Introduction

Deep venous thrombosis (DVT) is an established complication of all major and time dependent surgeries which may require immobilisation of a patient for a long period of time as in the case of many orthopaedic procedures and spinal surgeries^1,2^. Spinal surgeries may be lengthy, on certain frames and may involve patients with motor paralysis of lower limb which may have an association with Deep vein Thrombosis^1^.

Chemoprophylaxis against DVT and Pulmonary Embolism (PE) is being used in all surgical specialties except for spine surgery, when the fear of causing an unforgiving postoperative epidural hematoma may result in an irreversible neurologic disability for the patient^3-7^.

About 10-15% of the tribal population of India resides in Gujarat, particularly in South Gujarat. A high prevalence (13-31%) of sickle cell trait has been demonstrated among the different tribes of Gujarat. Venous thromboembolism is common in adults with sickle cell disease^8^. There is a gap in medical literature regarding incidence of thromboembolism in patients undergoing spine surgery, especially in South Gujarat region.

The purpose of this study was to demonstrate the incidence of venous thromboembolism in patients including those with sickle cell disease who underwent spine surgery and to formulate a protocol to provide prophylaxis to patients undergoing spine surgery in the future if the incidence was found to be significant with positive association between these two variables.

## Materials and Methods

A prospective non-randomised observational study was conducted at a Tertiary Health Setup in Surat between the period of January 2016 to October 2016 to assess the incidence of DVT in patients including those with sickle cell disease who underwent spine surgery. The purpose of the study was to determine if Sickle Cell Disease is a positive clinical predictor for Deep Venous Thrombosis in spinal surgery patients. All patients undergoing major spinal surgery at our institute, aged 18 years or more at time of surgery, of both genders, who accepted the terms set out in the study and signed the written informed consent form were included in the study. Patients with previous history of thromboembolic complications, on anticoagulant medication before surgery, peripheral vascular disease, diseases of the hematopoietic system, disseminated malignancy, surgery done under local anaesthesia and polytrauma patients were excluded. A total of 96 patients fulfilled the criteria and were enrolled in the study of which 17 patients were lost to follow-up and were excluded from statistical analysis.

Detailed history, clinical and neurological examination as per American Spinal Injury Association (ASIA) Grade, appropriate lab and radiological investigations were documented. Patient related data included: age, gender, body mass index (BMI), history of smoking, drug history (including contraceptives, hormonal therapy), pregnancy and other comorbidities. Surgery related data included diagnosis, level and type of the procedure, operative time, intra-operative blood loss, and duration of post-operative recumbency, post-operative neurological status which was also documented.

For radiological diagnosis of Deep Venous Thrombosis, Colour Doppler ultrasound was performed pre-operatively as well as on 7th and 14th post-operative day using 3-7 MHz convex probe on GE LOGIEQ F8 USG machine. All ultrasound procedures were done by a single radiologist to minimise inter-observer/subjective errors.

Sickling Test was conducted for all patients pre-operatively to screen them for Sickle Cell Disease. From each patient a complete hemogram (haemoglobin, platelets), coagulation profile [Prothrombin Time (PT), International Normalized Ratio (INR)], lipid profile, random blood sugar (RBS) was collected pre-operatively and 7th - 10th day post-operatively. D-dimer test was done in the post-operative period from 7th to 10th day.

During hospitalisation, patients were evaluated every day, in search of clinical changes consistent with thromboembolic events. In post-operative period mechanical prophylaxis (crepe bandaging in bilateral lower limbs) was given to every patient. Each patient was encouraged to mobilise from bed as soon as post-operative pain subsided and according to patient’s neurological status. Passive physiotherapy was given to all the patients with complete neurological injury. Pharmacological prophylaxis in form of Low Molecular Weight Heparin (LMWH), was provided to the patients, only when there was an evidence of Deep Venous Thrombosis on Doppler study. Outcomes in terms of incidence of Evidence of Deep Venous Thrombosis in spinal surgery patients was calculated and multiple binomial logistic regression analysis was conducted to find out association of various patient characteristic and sickle cell disease to that of the incidence of Deep Venous Thrombosis. The value of p<0.05 was taken to be significant in association of Deep Venous Thrombosis.

## Results

Seventy-nine patients who underwent spine surgeries and gave consent to be a part of this study, were included as study sample. The mean age of study group was 41 Years. Majority of patients belonged to 31-40 age group. 75% (n=59) patients included in the study sample were males (Table I). Only 8 patients (10.1%) were neurologically intact (ASIA grade E), while 12 patients (15.2%) had complete neurological injury (ASIA grade A).

**Table I:** Distribution of the study group according to age and sex

Age Group	Sex		Total
	Female	Male	
<30 Years	3	12	15 (19.0%)
31 - 40 Years	8	20	28 (35.4%)
41 - 50 Years	5	12	17 (21.5%)
51 - 60 Years	2	11	13 (16.5%)
61 - 70 Years	2	4	6 (7.6%)
Total (% with SEX)	20 (25.32%)	59 (74.68%)	79 (100.0%)

All 79 patients had normal venous Doppler pre-operatively. Two patients (2.5%) developed evidence of deep vein thrombosis in bilateral lower limbs on Doppler ultrasound done on 7th and 14th post-operative day. Two patients (2.5%) had evidence of venous stasis on Doppler ultrasound with sluggish flow in anterior tibial vein (bilateral), but no evidence of thrombosis (Table II). None of the patients had shown clinical signs of Deep Venous Thrombosis in daily post-operative clinical examination of the patient.

**Table II:** Distribution of patients according to Doppler ultrasound results

Doppler ultrasound	Pre-operative	Post-operative (7th day)	Post-operative (14th day)
Normal	79	75	75
DVT	0	2 (2.5%)	2 (2.5%)
Venous Stasis	0	2 (2.5%)	2 (2.5%)

One patient developed pulmonary embolism (PE). The patient was managed in ICU with mechanical ventilatory support and subcutaneous Low Molecular Weight Heparin (LMWH). Patient was extubated within two days and recovered well. Patient was a known case of Diabetes Mellitus (DM-Type2) and hypertension, under regular treatment.

Total 9 patients (11.4%) out of 79 patients were sickle cell positive from which 4 patients showed evidence of Deep Venous Thrombosis or Venous Stasis post-operatively. The association of sickle cell disease as risk factor for Deep venous thrombosis was statistically significant in-patient undergoing spinal surgeries, (Fischer exact test after Haldane Anscombe correction, p value<0.002)

69 patients (87%) patients were mobilised within 5 days of surgery, of which 46 patients (58%) were mobilised within 3 days of surgery. Many patients (n=25) were mobilised on the next day of surgery. Two patients diagnosed with Deep Venous Thrombosis later on, were also mobilised on 1st post-op day and were operated for lumbar disc pathology. Two patients who were diagnosed with venous stasis on 7th post op day were mobilised on 3rd post op day. We could not find any statically significant association with the incidence of Deep Venous thrombosis with the period of immobilisation. Also, all the patients irrespective of the period of immobilisation were given mechanical prophylaxis.

Patients with ASIA grade D and E were advised to walk with brace with/without assistance. Patients with ASIA grade A, B and C were advised to log roll and start wheel-chair mobilisation as soon as their post-operative pain subsided. All the patients and their relatives were advised and taught passive and/or active mobilisation of peripheral limbs and joints under supervision of a physical therapist.

Thirty-six patients (45.6%) were operated on for pathology in lumbar spine, 21 patients (26.6%) for cervical spine and 22 patients (27.8%) for thoracic spine pathology. All the 4 patients having evidence of thrombosis/venous stasis in this study were operated on lumbar spine. Of the two patients who had Deep Venous Thrombosis, one patient had an infective aetiology while the other patient had a degenerative aetiology.

D-Dimer test could be performed only on 60 patients due to unavailability of reagent at a particular given time for the remainder patients. It was positive in 5 (8.3%) patients out of 60 patients. Out of these five patients, two patients had Deep Venous Thrombosis and two patients had sluggish venous blood flow (venous stasis) in lower limbs.

All patients in our study were ‘At Risk’ (moderate, high, or highest) for thromboembolic complications. Two patients with positive Doppler ultrasound finding for Deep Venous Thrombosis were from high-risk group. For patients with venous stasis, one patient belonged to moderate risk group while the other one belonged to highest risk group (Table III and IV).

**Table III:** Distribution of patients according to risk factors (to segregate the patients in high, intermediate and low risk group for DVT)

Sr. No.	Risk factors for DVT	Point	No. of patients	Percentage (%)
1	Overweight and obese (BMI>25)	1	10	12.65
2	Age 41-60 year	1	30	39.97
3	Age 61-75 year	2	05	6.32
4	Confined to bed >72hour	2	11	13.92
5	Malignancy within 5 years	2	0	0
6	Major surgery (>45min)	2	79	100
7	Age (>75year)	3	0	0
8	History of DVT	3	0	0
9	Spinal injury/Paraplegia/quadriplegia	5	24	30.37

**Table IV:** Distribution of patients according to the risk groups

Point	0-1	2	3-4	5+
Risk group	Low risk	Moderate risk	High risk	Highest risk
No. Of patients among groups (out of 79 patients)	0	26	29	24
Percentage (out of 100)	0%	32.91%	36.71%	30.38%

## Discussion

DVT and PE caused by long period of immobilisation are life threatening medical complications following all major orthopaedic surgeries. Surgery of any kind is known to create prothrombotic state due to stress which may increase thrombotic complications post-operatively^9^. The incidence of Deep Venous Thrombosis after spine surgery ranges from 0.3% to 31% and further studies are needed for the same. The reason for this variability in the incidence of Deep Venous Thrombosis is most likely due to the differences in epidemiological characteristics of the studied populations, lack of standard diagnostic and treatment protocol for DVT^1,2,10^.

Takenori Oda *et al* documented Deep Venous Thrombosis venographically in 5.6% patients undergoing cervical procedures, and 26.5% patients undergoing lumbar procedures in a prospective study to evaluate Deep Venous Thrombosis after posterior spinal surgery^11^.

A Cohort study was done by Sreedharan Namboothiri in 2012 where a prospective evaluation of 121 patients who underwent major spine surgeries was conducted to find the incidence of only clinically identifiable Deep Venous Thrombosis. The incidence of Deep Venous Thrombosis was only 0.78% and did not show statistically significant correlation between incidence of Deep Venous Thrombosis and spine surgery^2^.

In our study, the incidence of Deep Venous Thrombosis was 2.5%, of which one patient developed symptomatic pulmonary embolism. Two patients (2.5%) had venous stasis on venous doppler, but there was no evidence of thrombosis. No patient developed clinical signs of Deep Venous Thrombosis. All the 4 patients with abnormal Doppler ultrasound findings were positive for D-Dimer and had undergone surgeries for lumbar spine. There was no mortality due to thromboembolic complications.

Clinician cannot only rely on clinical signs of Deep Venous Thrombosis for the diagnosis. In our study, none of the patients with positive Doppler ultrasound had any clinical signs of Deep Venous Thrombosis. Both the patients were diagnosed with Deep Venous Thrombosis on 7th postoperative day. During this period, patients might develop fatal complications of venous thromboembolism^12^. D-Dimer test is a reliable indicator for risk of Deep Venous Thrombosis. Patients with potential risk factors for Deep Venous Thrombosis should be investigated for D-Dimer as early as possible in post-operative period^13,14^.

Prophylaxis consists of mechanical and pharmacological measures which has been most effective for venous thromboembolism as demonstrated in several previous studies^3-6,12,15,16^. Mechanical prophylaxis (compression stocking or sequential compression devices) after spine surgery is considered both effective and safe but chemoprophylaxis with anticoagulation therapy is not accepted to be safe as early anticoagulation chemoprophylaxis has the potential risk for bleeding complications, specifically, acute post-operative formation of epidural haematomas which has significant neurological sequalae^17,18^. Timing for starting chemoprophylaxis postoperatively is also controversial as there is no conclusive evidence that exists in the literature to guide management for such patients^3,5,6,7,15,18^. In our study, all the patients who underwent spinal surgeries were given mechanical prophylaxis to mitigate the risk of epidural haematoma associated with use of pharmacological prophylaxis.

Multiple risk factors are responsible for the development of Deep Venous Thrombosis in addition with spine surgery but for segregation of patients pre-operatively into risk group there is still no universal criteria. Thair M Al-Dujaili *et al*^1^ in a prospective study of 158 patients who underwent spinal surgical procedures attempted to segregate patients according to risk profiles into risk groups pre-operatively. The American College of Chest Physicians Consensus Conference on Antithrombotic and Thrombolytic Therapy listed the major risk factors for development of Deep Venous Thrombosis. Joseph Caprini quantified these factors in a reproducible manner to help surgeons in evaluating preoperative risk-factor assessment^7^.

In the present study, risk factors for each patient were calculated to produce an overall risk factor score according to Caprini s risk factor score, which corresponded from low to very high potential for development of Deep Venous Thrombosis. A total of 67.7% of the patients in this study had at least one or more additional risk factors including age, bed confinement, history of malignancy, history of Deep Venous Thrombosis, obesity, and spinal cord injury with paralysis. On reviewing the risk factors for Deep Venous Thrombosis development in the studied population, 31.6% were at moderate risk, while 55% were at high risk, and 13.4% were at the highest risk. Risk of Deep Venous Thrombosis increases as age advances, and it is 3 times more common after the age of 75 years^9^. Also, the risk of Deep Venous thrombosis has been more in female sex^9^.

In our study, majority of the study sample (>50%) had age less than 40 yrs. 39.2% (31 patients) belonged to age group of 41 - 60 years. Patients who developed Deep Venous Thrombosis were equally distributed in younger (<40 years) and older age (>60 years) groups and all were females. So, risk of Deep Venous Thrombosis cannot be underestimated in younger age group. Incidence of Deep Venous Thrombosis was more among females in our study which is consistent with studies in literature implying positive correlation of association of gender with incidence of Deep Venous Thrombosis in patients undergoing spinal surgeries.

In spinal trauma, neurological deficit causes venous stasis which may lead to activation of the Virchow’s triad leading to increased risk of Deep Venous Thrombosis^18^. In our study, majority of the patients (44% patients) belonged to trauma group patients but contrary to the previous studies, the 4 patients with evidence of Deep Venous Thrombosis / venous stasis belonged to infective or degenerative etiological category.

Patients with spinal cord injuries especially with ASIA grade A or B are at highest risk of Deep Venous Thrombosis^18^. But in our study, none of the patients within this group (n = 24 patients) developed clinical signs or evidence of thrombosis on serial colour Doppler ultrasound. Around half of the patients were operated within 2 days of primary insult, and 90% of the patients were mobilised within 3 to 5 days postoperatively. Mechanical prophylaxis was provided to all patients.

Pulmonary hypertension, infection, acute kidney injury and cerebrovascular stroke are life-threatening complications in sickle cell disease. Patients with Sickle Cell Disease also have higher risks of vaso-occlusive crisis, acute chest syndrome. Pro-thrombotic state occurs in sickle cell disease patients by various mechanisms such as erythrocyte adhesion, endothelial dysfunction, leukocyte activation, platelet aggregation, coagulation defects, and free haemoglobin-induced oxidative damage. In sickle cell disease nitric oxide scavenging secondary to intravascular haemolysis also has been hypothesised to cause hypercoagulable state which may lead to complications like Deep Venous Thrombosis^19-23^.

In our study, 11.4% patients (n=9) were diagnosed with sickle cell disease. All the 4 patients with evidence of thrombosis or stasis on Doppler study were positive for sickle cell disease indicating positive association of sickle cell disease with incidence of Deep Venous Thrombosis. For a patient with sickle cell disease a careful pre-operative check must include an assessment of the patient's known crisis triggers, hematologic profile, evaluation of risk of surgical procedure and anticipated volume of blood loss, period of immobilisation after surgery and opioid dependence. Hypoxia, hypothermia, acidosis, and intravascular volume depletion should be prevented in such sickle cell patients in the perioperative period^24^.

Drawbacks of this study includes the limited available sample size and the lack of advanced investigations like high performance liquid chromatography (HPLC) and electrophoretic study which would have helped in finding out the type of sickle cell disease (trait or disease) and its association with the incidence of Deep Vein Thrombosis.

## Conclusion

The findings of this study report the incidence of Deep Vein Thrombosis in patients undergoing spine surgeries to be 2.5%. Also, 2.5% of patients had venous stasis on Doppler ultrasound but no evidence of thrombosis. Clinician should have a higher index of suspicion for Deep Vein Thrombosis in patient with sickle cell disease undergoing spinal surgeries. Post-operative D-dimer measurements in patients from high-risk group who are undergoing spine surgery, may provide a complementary diagnostic screening for Deep Venous Thrombosis during early post-operative days. Mechanical prophylaxis should be provided to all patients who undergoing spine surgery. Pharmacological prophylaxis should be reserved for patients showing D-dimer positive and/ or Doppler findings positive.
